# Eco‐Conscious RP‐HPLC Method for Concurrent Quantification of Assay and Dissolution Testing of Methocarbamol and Diclofenac Potassium in Tablet Formulations

**DOI:** 10.1155/ianc/8947770

**Published:** 2026-04-21

**Authors:** Hedia Ouni, Fahad M. Alminderej, Mostafa E. Salem, Sayed M. Saleh, Omkulthom Al kamaly, Mahmoud A. Mohamed

**Affiliations:** ^1^ Department of Chemistry, College of Science in Yanbu, Taibah University, Yanbu Governorate, Saudi Arabia, taibahu.edu.sa; ^2^ Department of Chemistry, College of Science, Qassim University, Buraidah, 51452, Saudi Arabia, qu.edu.sa; ^3^ Department of Chemistry, College of Science, Imam Mohammad Ibn Saud Islamic University (IMSIU), Riyadh, 11623, Saudi Arabia, imamu.edu.sa; ^4^ Department of Pharmaceutical Sciences, College of Pharmacy, Princess Nourah bint Abdulrahman University, P.O. Box 84428, Riyadh, 11671, Saudi Arabia, pnu.edu.sa; ^5^ Hikma Pharmaceutical Company, Beni-Suef, 62511, Egypt

**Keywords:** Analytical Eco-Scale, anti-inflammatory and analgesic effects, assay and dissolution testing, CACI, diclofenac K, EPPI, methocarbamol, RP-HPLC

## Abstract

A common combination of methocarbamol (MTH) and diclofenac potassium (DCL K) relieves musculoskeletal pain and inflammation through centrally acting muscle relaxants. A dual‐function RP‐HPLC method was used to analyze the assay and dissolution testing of MTH and DCL K simultaneously. As part of the suggested method, green and white analytical chemistry standards were incorporated to ensure sustainability and compatibility. We performed a chromatographic separation on a C8 column (15 cm × 4.6 mm, 5 μm) with a mobile phase of phosphate buffer and ethanol (30:70 *v*/*v*), adjusted to pH 2.5 ± 0.1, yielding sharp and well‐resolved peaks with retention times of 2.183 min for MTH and 6.652 min for DCL. A 275‐nm dual UV detector was used to detect the separation at a flow rate of 1.0 mL/min. Sensitivity parameters demonstrated low detection capabilities, with limits of detection (LOD) of 0.069 μg/mL for MTH and 0.012 μg/mL for DCL, and limits of quantification (LOQ) of 0.210 μg/mL and 0.035 μg/mL. Over an extended sample period of 30 min with 100 revolutions per minute (rpm), USP Apparatus II (Paddle) was used to measure dissolution at a temperature of 37.0 ± 0.5°C in phosphate buffer (pH 6.8). With both active pharmaceutical ingredients, 75% of the drug was released within 30 min. A combination of suitability (RSD 2%) and precision (raw data 2%) makes the method an excellent choice for routine quality control. In keeping with the changing environment and regulatory requirements, pharmaceutical analysis can become more efficient and sustainable. Accelerated stability studies were conducted on the finished dosage form under ICH‐recommended conditions for 6 months. Sustainability was assessed using the Analytical Eco‐Scale, Environmental and Practical Performance Index (EPPI), and Click Analytical Chemistry Index (CACI). The method achieved an Eco‐Scale score of 91, classifying it as an excellent green method, an EPPI score of 97.9, and a CACI score of 80, confirming its efficiency and practicality for routine quality control.

## 1. Introduction

Musculoskeletal pain is commonly treated with methocarbamol (MTH), a centrally acting skeletal muscle relaxant, in combination with DCL. The most commonly used drug for treating muscle spasms is MTH. It depresses the central nervous system, thereby acting as a relaxant for skeletal muscles. As a combined preparation for pain relief, MTH is given with analgesics. A combination of MTH and DCL is a famous nonsteroidal anti‐inflammatory drug [1].

An antispasmodic drug known for treating intense skeletal muscle spasms, MTH is powerful even in the most severe cases (Figure 1(a)). The medication lessens upper motor neuron spasms similar to those caused by dantrolene and baclofen. MTH may block the midbrain reticular activating system. There is a well‐known mechanism by which anticholinergic drugs work. Consequently, muscle tone and polysynaptic reflexes become much weaker. Interneurons in the spinal cord are indirectly modulated by this process. MTH does not directly affect skeletal muscle or motor nerve fiber contractility. A highly effective medication, MTH can be taken orally and is highly effective within 30 min of administration. As soon as 2 hours have passed since the medication was administered, the plasma concentration peaks. This compound is metabolized by the liver, and its half‐life is approximately one to 2 hours. This drug is primarily eliminated through renal excretion as an inactive metabolite. MTH has been shown to be beneficial when co‐administrated with diclofenac K or Na in numerous studies, elucidating their effectiveness in relaxing muscles, relieving pain and inflammation, including redness and swelling, and improving muscle mobility [2].

FIGURE 1Chemical structure of (a) MTH and (b) DCL K.

**Figure:**
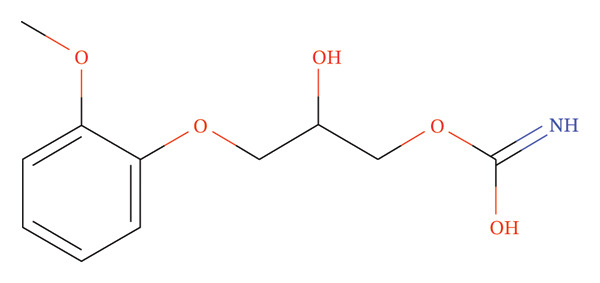
(a)

**Figure:**
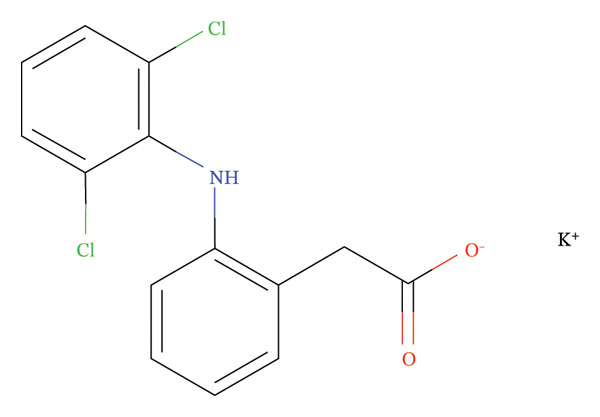
(b)

A breakthrough shift toward chemical processes that are ecologically conscious and sustainable has been achieved through the implementation of green chemistry principles. Having a good understanding of the 12 Regulations of Green Chemistry is crucial for analysts in order to reduce environmental waste and achieve sustainability [3, 4]. An integral part of green chemistry is the integration of method validation criteria to assess the environmental sustainability of analytical techniques. In addition to increasing public awareness of analytical chemistry, these criteria also demonstrate a methodology’s long‐term viability [5, 6]. The implementation of green chemistry initiatives is therefore essential for the shaping of a sustainable future [7, 8]. Chromatographic methods have been advanced in large part because of sustainability. A careful assessment of their potential environmental impact is required to ensure HPLC techniques benefit the environment [9–12].

The K salt of DCL is Potassium [2‐(2,6‐dichloroanilino)phenyl]acetate (Figure 1(b)). Through its interference with prostaglandin synthetase, it inhibits prostaglandin synthesis, a powerful analgesic, anti‐inflammatory, and antipyretic [13].

The determination of these drugs singly and as a combination has been reported in different spectroscopic and chromatographic methods. Recent spectroscopic strategies have utilized reagent‐based color growth and molecular spectrophotometric analyses to quantify related analytes using a satisfactory sensitivity and working simplicity, which illustrates the continued validity of cost‐effective spectroscopic tests in ordinary labs [14]. Besides these, chromatographic techniques, in particular, reversed‐phase high‐performance liquid chromatography (RP‐HPLC), have been developed fully to maximize resolution of the peaks, reduce the length of the analysis, and offer the stability of the system of multi‐analyte systems; the common improvements include optimization of mobile phase composition, pH, and ionic strength, as well as column chemistry, in an effort to ensure that the system separates the baseline and is stable enough to work in the specific concentration of interest [15]. Despite these being done, possibilities still exist to optimize the run time with such a high level of linearity, precision, and sensitivity and explicitly report figures of method performance, including retention times, limits of detection (LOD), and limits of quantification (LOQ), which are useful in the proper profiling of drug release and monitoring the effects of formulation variables. Besides the measurement of final products, HPLC is central to the dissolution testing, and in this regard, the selectivity of the methodology, stability‐indicating performance, and time resolution are essential in the appropriate profiling of drug release and observing the variations of formulation variables. Contemporary reviews highlight the best practices in the combination of dissolution procedures and HPLC detection, which ought to be added to guarantee the soundness of data and performance of regulations, including the choice of the correct media, methods of filtration, and elements of validation, including correctness, accuracy, and steadiness of the solution [16]. With these principles in the design of the methods, a stability‐indicating RP‐HPLC method can be obtained that can facilitate the assays and dissolution of both MTH/DCL and thus enable the analysis process to be more efficient in quality control settings. Analyzing pharmaceutical chemicals alone, in combination with other medications, or in biological fluids has also been developed using a variety of analytical methods. A number of these methodologies include HPLC and UPLC [17–20].

The presented RP‐HPLC methodology is a novel and ecologically friendly analytical technique that fills the critical loopholes within the MTH and diclofenac potassium (DCL K) analysis techniques published in the past. Unlike conventional designs, where most commonly assay or dissolution analyses are carried out, our design is designed to be a dual‐purpose platform to enable the determination of both parameters within the same validated procedure. It is extremely helpful and saves a lot of time in analysis, expenses, and resources, which is why it is of paramount concern in standard quality control in the pharmaceutical industries. The key feature of this method is that it is an eco‐friendly approach and is related to the notions of Green Analytical Chemistry (GAC) and White Analytical Chemistry (WAC). Incorporation of safe, renewable, and less toxic solvents such as ethanol to replace widely used toxic organic solvents such as acetonitrile or methanol will lead to less EI and lower risk for those handling the procedure. Second, chromatographic parameters are optimized systematically, using C8 columns and an isocratic mobile phase of phosphate buffer and ethanol (30:70 *v*/*v*) at pH 2.5, providing high‐quality resolution without a complex gradient program or a high‐cost stationary phase. It has also been observed that the approach is highly regulatory and practical. It is USP dissolution compliant; more than 75% of the drug is dissolved after 30 min under standard conditions and it has good precision and system suitability (RSD ≤ 2%). Its use in quality control laboratories ensures its strength and durability. This is, as far as we can determine, the first reported eco‐friendly RP‐HPLC method that also permits simultaneous testing of assays and the dissolution of both MTH and DCL K in combined tablet preparations. As a sustainable and analytically accurate solution, the given solution is a valuable innovation in pharmaceutical analysis. It encourages more efficient resource‐based practices across the globe.

## 2. Experimental Section

### 2.1. Chemicals and Reagents

A high‐quality analytical or HPLC‐grade range of chemicals and reagents was used throughout this study. Ethanol (HPLC grade) was obtained from Merck (Darmstadt, Germany). Sodium hydroxide and orthophosphoric acid were obtained from Sigma‐Aldrich (St. Louis, MO, USA). Sigma‐Aldrich provided sodium monobasic phosphate and potassium dihydrogen phosphate as well. Throughout the experiment, deionized water was consumed, and no further purification was performed.

### 2.2. Instruments

An HPLC system with a quaternary pump, autosampler, and UV detector (Waters Alliance e2695, Waters Corporation, Milford, MA, USA) was used to perform chromatographic analysis. The dissolution tests were conducted on a USP Dissolution Apparatus II (Paddle type) (Pharmatest PTWS 120D, Hainburg, Germany). Empower 3 Chromatography Data Software was used to perform data acquisition, processing, and reporting.

### 2.3. Chromatographic System

The separation was done using chromatography in Inertsil C8 HPLC columns (15 cm × 4.6 mm, 5 μm particle size) with a pore size of 150 Å. The mobile stage was a mixture of phosphate buffer and ethanol in the proportion of 30:70 (*v*/*v*) with a pH of 2.5 ± 0.1 adjusted using orthophosphoric acid. It was permitted a flow rate of 1.0 mL/min and detected with a dual UV detector at 275 nm. Injection was done in 20 μL of volume, and the column temperature was maintained at 25°C in the isocratic mode. Each analysis took 9 min of running time. All the solutions were filtered using a 0.45 μm membrane filter and degassed.

### 2.4. Diluent

Use the mobile phase as a solvent.

### 2.5. Stock and Working Standard Solutions

An appropriate quantity of 50 mg of DCL K and 500 mg of MTH working standards were transferred into a 100‐mL volumetric flask. After adding 70 mL of diluent, the mixture was sonicated until fully dissolved. A similar diluent was used to adjust the mixture to the mark. To dilute the solution in the mobile phase, 5 mL was added to a 500‐mL volumetric flask. Before injecting the solution, it was filtered through a syringe filter (Figure 2(a)). The final concentrations were 5 μg/mL of DIC and 50 μg/mL of MTH.

FIGURE 2Chromatograms for the assay test (a) standard and (b) tablet sample solution.

**Figure:**
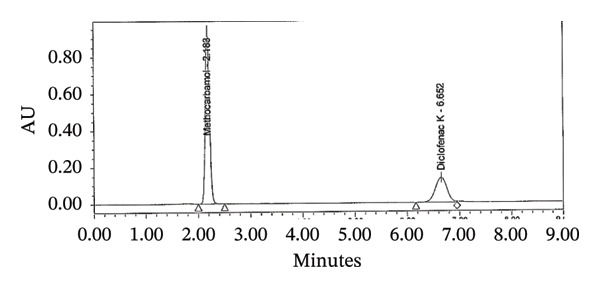
(a)

**Figure:**
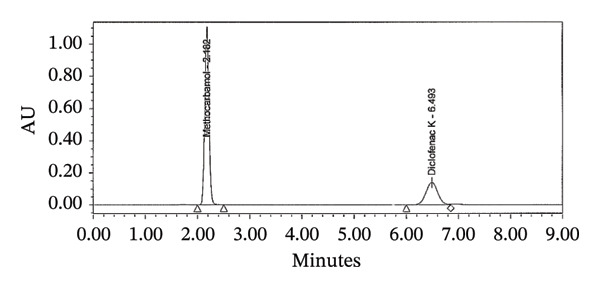
(b)

### 2.6. Analysis of Dosage Formulations

A sample of Flexilax 50/500 mg film‐coated tablet (FCT) batch number #274 was prepared by finely powdering 20 tablets (calculating the average weight). In a 100‐mL volumetric flask, 70 mL of a diluent was added. The flask was filled with 850 mg of powder. After 30 min of sonication and dilution with the same diluent, the sample solution was dissolved thoroughly. Diluent was used to dilute 5 mL into a 500‐mL volumetric flask. The solution was then filtered through a syringe filter and injected (Figure 2(b)). The final concentration was 5 μg/mL of DIC and 50 μg/mL of MTH.

### 2.7. Establishment of Calibration Curves

Calibration curves were prepared by making a series of standard solutions of DCL K and MTH at various concentration levels within the desired analysis range. All the solutions were individually injected three times under the stated chromatographic conditions, and the relative peak areas were noted. An analysis of peak area versus concentration of each analyte was performed, followed by the development of a linear regression model that predicted the calibration equation and correlation coefficient (*r* ^2^).

### 2.8. Dissolution Media and Conditions

Dissolution testing was conducted on a USP Apparatus II (Paddle type) rotating at a constant speed of 100 revolutions per minute (rpm). The dissolution medium was a mixture of 900 mL of phosphate buffer (pH 6.8) and kept at 37.0 ± 0.5°C. The test lasted 30 min. To prepare the phosphate buffer (pH 6.8), 6.8 g of potassium dihydrogen phosphate were mixed with 0.89 g of sodium hydroxide in a 1000‐mL volumetric flask. With orthophosphoric acid or sodium hydroxide, the pH was adjusted to 6.8.

### 2.9. Preparation of Standard Solution for Dissolution Tests

The standard solution was prepared using 50 mg DCL K working standard and 500 mg MTH weighed appropriately and placed in a 100‐mL volumetric flask. The mixture was sonicated until fully dissolved after adding 70 mL of mobile phase. It was then diluted to volume with the same mobile phase. By pipetting 5 mL into a 500‐mL volumetric flask and diluting with dissolution medium, the solution was diluted to volume. An appropriate syringe filter was used to filter the final solution for analysis (Figure 3(a)).

FIGURE 3Chromatograms for the dissolution test (a) standard and (b) tablet sample solution.

**Figure:**
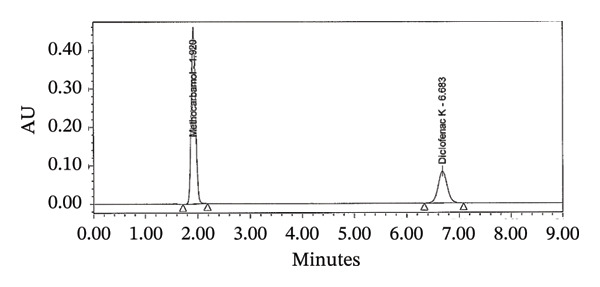
(a)

**Figure:**
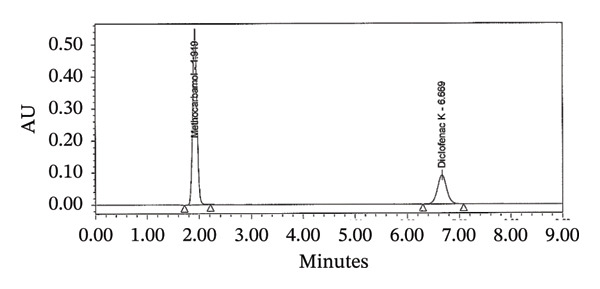
(b)

### 2.10. Preparation of Samples for Dissolution Tests

In the case of a dissolution test, 900 mL of dissolution medium at 37 ± 0.5°C was placed in each vessel. Each tablet was dropped into each cup, and equipment was used for 30 min under the indicated conditions. The test was completed and 20 mL of the solution in each vessel was filtered through a suitable syringe filter before analysis. In this solution, 1 mL was pipetted into a 10‐mL volumetric flask and diluted to volume using dissolution medium (Figure 3(b)).

### 2.11. Assessment of Environmental Sustainability Using Analytical Tools

#### 2.11.1. The Click Analytical Chemistry Index (CACI) Tool

The CACI is an up‐to‐date and easy‐to‐use instrument used to assess analytical methods based on their simplicity, efficiency, and practicality. Through a perspective grounded in click chemistry, CACI is an innovative approach to method assessment. It offers a quantitative measure and open‐source software accessible at bit.ly/CACI2025. Its major advantages are the objective comparison of methods. It also promotes innovative and sustainable methods and promotes the development of methods that can be easily applied to regular laboratory practice. There are some limitations to CACI. In addition, the framework is limited in scope compared to other validation frameworks, scoring is subject to subjectivity, and regulatory application is not possible. It has been suggested that CACI applications should be implemented with well‐known standards, like ICH Q2, and common input parameters customized to specific analysis areas, and users should participate in the software development process. It could also be extended to promote CACI application and make it a part of green chemistry measurements. CACI is a powerful screening and decision‐making tool that complements other traditional techniques. It allows researchers to create analytical techniques that are relevant, viable, and sustainable [21].

### 2.12. Analytical Eco‐Scale Tool

The Analytical Eco‐Scale is a semi‐quantitative instrument to assess the impact of analytical procedures on the environment according to GAC concepts. It is based on a basic scoring system where the most efficient green procedure begins with 100 points. Penal points are subtracted concerning aspects that adversely impact sustainability, which include the use of toxic chemicals, excessive energy use, and the generation of unwanted waste materials. The score is essentially obtained as Eco‐Scale Score = 100 minus the number of total penalty points, and the method can be labeled *Excellent* (> 75 points), *Acceptable* (50–75 points), or Inadequate (< 50 points). In addition to providing a convenient, objective comparison of contrasting analytical processes, this method can reduce solvent usage, energy consumption, and the need for reagents to be disposed of [22].

### 2.13. The Environmental and Practical Performance Index (EPPI) Tool

The EPPI is a twofold, combined index that measures (i) an Environmental Index (EI), which represents the greenness of the sample preparation and measurement, and (ii) a Performance and Practicality (PPI) component that represents analytical performance and applicability. The EPPI will yield a score (1–100) and provide a pie chart that separates (visually) environmental (green) and performance/practicality contributions (purple), allowing for a more balanced, so‐called white viewpoint that is compatible with the most current sustainability paradigms [23].

### 2.14. Accelerated Stability Study

A stress test on a dosage form was performed to assess the stability of the dosage form under stress conditions. This was recommended by ICH guidelines. Samples were maintained at 40°C ± 2°C/75% RH ± 5% at 6 months, and assessments were done at fixed time intervals of 0, 1, 3, and 6 months. In addition, the assay and dissolution performance of the samples were evaluated using the validated HPLC procedures and the USP dissolution apparatus at every interval in the samples. Assay was established through determination of the active pharmaceutical constituents against prepared fresh standards, and dissolution was determined under certain conditions (USP II, 900 mL phosphate buffer pH 6.8, 100 rpm, 37°C ± 0.5°C). To determine whether there was a USP potency or release profile change, the results were compared with the initial values [24].

## 3. Results and Discussion

### 3.1. Development and Optimization of the Method

The design of an RP‐HPLC procedure to simultaneously determine MTH and DCL in multicomponent tablet mixtures was informed by the goals of attaining high specificity, a short analysis duration, and environmental sustainability. Various chromatographic variables were carefully selected, including stationary phase, mobile phase, pH, and measurement wavelength.

### 3.2. Choice of Stationary Phases

Reversed‐phase HPLC: The selection of either a C18 or C8 column is largely determined by analyte hydrophobicity, required retention strength, and optimal peak shape and analysis time. C18 phases have greater hydrophobic interactions and longer retention times, usually because of increased carbon load and surface hydrophobicity. The literature of present greenness‐oriented chromatographic techniques emphasizes that extremely hydrophobic phases may be unnecessary since they are able to significantly extend the length of run times and can consume a lot of solvent, which makes the method less sustainable and feasible in analysis processes. On the other extreme, C8 columns provide intermediate hydrophobic retention; hence, they are quite appropriate with analytes of intermediate polarity, including MTH and DCL K. To enhance an even better balance of elution strength with greener mobile phases (e.g., with smaller concentrations of strong organic modifiers), reduction of stationary‐phase hydrophobicity is widely used. Recent literature on sustainability highlights that optimizing column chemistry to reduce solvent use and minimize chromatographic run time is a key approach for developing performance‐optimized and environmentally conscious procedures. Preliminary experiments using C18 columns in our approach development resulted in excessively long retention times, overly wide peaks, and higher solvent consumption. The shift to a C8 column (150 mm × 4.6 mm, 5 μm) led to much better performance with shorter retention times, more defined peaks of both analytes, and the method being more robust, though at the same time preserving high resolution. These results are in line with the documented literature that proposes that the method’s greenness and practicality are improved by picking stationary phases that minimize analysis time and resource consumption without affecting the quality of the analysis. C18 and C8 columns were first tested to determine which stationary phase was appropriate. C18 gave an excellent retention but also caused increased run times and backpressure. The C8 column (150 mm × 4.6 mm, 5 μm) provided the most efficient compromise between analysis time and resolution, so it was the most appropriate option.

### 3.3. Mobile Phase Composition and Green Solvent Selection

To ensure that things are in line with GAC, ethanol was used as the organic modifier rather than the usual acetone solvents (acetonitrile) or methanol, which are toxic and hazardous to the environment. Different combinations of phosphate buffer (pH 2.5) and ethanol were tested (20:80, 30:70, and 40:60 *V*/*V*). A 30:70 (buffer: ethanol) ratio delivered sharp and symmetrical peaks with significant retention factors for the two analytes.

### 3.4. pH Optimization

The aqueous phase pH was the key to gaining maximum symmetry and reducing tailing. Experiments were done at a pH of 2.0–3.5. Optimal peak shape and resolution were observed at a pH of 2.5 ± 0.1 without affecting column stability.

### 3.5. Detection Wavelength

The UV spectra of the two drugs were suspended at 200–400 nm, and a common wavelength of 275 nm was chosen to be scanned concurrently, giving sufficient sensitivity to both analytes.

### 3.6. Flow Rate and Suitability of the System

Flows of 0.8–1.2 mL/min were investigated. Flow rates of 1.0 mL/min were the best rates to balance resolution, analysis time, and column backpressure. In such optimal conditions, the method was found to have acceptable system suitability parameters such as the theoretical plate, tailing factor, and percent RSD. These parameters had acceptable limits.

The last optimized technique did not only provide strong separation and quantification of MTH and DCL but also proved sustainable by decreasing the use of hazardous solvents, which is why this technique meets sustainability objectives in pharmaceutical analysis.

### 3.7. Method Validation

The developed HPLC method was thoroughly validated for the dual determination of MTH and DCL in fixed dose combination tablets. The validation process was conducted in strict accordance with ICH guidelines to ensure the method’s validity [25].

### 3.8. Linearity

Linearity of the proposed RP‐HPLC methodology was determined to ascertain its capacity to give results that are directly proportional to the concentration of MTH and DCL in a given range. Both analytes were prepared as standard stock solutions serially diluted to give five concentration levels of the desired assay concentration. In the case of MTH, the concentration was 3–70 μg/mL, and DCL was 1–10 μg/mL.

Triplicate injections of each concentration level were done and the relevant peak areas plotted against the respective concentrations to establish calibration curves. Both drugs showed significant linearity in the method with correlation coefficients (*R* ^2^) of more than 0.999, signifying a significant linear relationship. The regression models were

(1) MTH: y=a1x+b1,

(2) DCL: y=a2x+b2.

*Y* is the area of the peak, and *x* is the concentration in μg/mL. The value of the intercept was low, and there were no significant residual plots that indicated the lack of significant systematic error. These findings show that the procedure is appropriate for the correct quantification of both the analytes at the tested concentration (Table 1).

**TABLE 1:** Calculation of calibration curve parameters and DCL and MTH regression analysis.

Drug	MTH	DCL
Wavelength (nm)	275 nm	275 nm
Range (μg/mL)	3–70	1–10
Coefficients of determination (*R* ^2^)	0.9999	0.9999
Slope	10,552.8068	42,281.0173
Intercept	−184.6646	−542.2312
*S*_*y*/*x*_	722.8881	312.0309
Sa	221.7290	150.0391
Sb	11.4329	38.7399
LOD^a^ (μg/mL)	0.069	0.012
LOQ^b^ (μg/mL)	0.210	0.035

^a^Limit of detection (3.3 × *σ*/slope) and.

^b^Limit of quantitation (10 × *σ*/slope).

### 3.9. LOD and LOQ

An RP‐HPLC method demonstrating sensitivity was developed by measuring the MTH and DCL LOD and LOQ. Calculations were made using ICH‐recommended equations based on the standard deviation of the response and slope of the calibration curve:

(3) LOD=3.3×σS,

(4) LOQ=10×σS,

where *σ* = standard deviation of the regression line y‐intercepts and *S* = the slope of the calibration curve.

The calculated LOD and LOQ values demonstrated the high sensitivity of the method. This allowed reliable detection and quantification of both analytes at low concentration levels (Table 1).

### 3.10. Precision

A repeatability and intermediate precision evaluation was carried out to evaluate the precision of RP‐HPLC. MTH and DCL were injected in six replicates at the same concentration under the same operating conditions to assess repeatability. Different analysts used the same equipment and performed the analyses on different days to determine intermediate precision. %RSD is a measure of relative standard deviation. Based on ICH Q2(R2) guidelines, both analytes had less than 2% RSD, which is within acceptable limits. Consequently, the method is precise and suitable for routine quality control (Table 1).

### 3.11. Accuracy and Recovery

Recovery studies were conducted at three concentration levels of the target assay concentration to evaluate the accuracy of the test. A placebo matrix was spiked with known quantities of MTH and DCL, and the proposed method was used to analyze the results. Triplicates of each level were tested, and percentage recovery rates were calculated. As both analytes were recovered within the acceptable range of 98%–102%, the method demonstrated its accuracy and reliability in quantifying active ingredients in tablet formulations (Table 2).

**TABLE 2:** Results of the accuracy and recovery of the MTH and DCL drug analysis.

Parameters	HPLC	
Relative concentrations %	MTH	DCL
	Recovery (%)	Recovery (%)
20	98.23	99.45
	99.56	98.97
	100.11	100.09
	100.36	99.63
	99.44	99.49
	99.78	99.65

Mean ± RSD	99.85% ± 0.38	99.57% ± 0.40
80	99.58	100.16
	99.78	98.49
	101.25	99.41
	99.37	100.48
	98.40	99.26
	99.67	99.08

Mean ± RSD	99.69% ± 1.02	99.34% ± 0.73
120	100.20	100.16
	99.48	98.35
	98.18	100.17
	100.41	98.48
	99.58	100.27
	99.39	100.08

Mean ± RSD	99.41% ± 0.80	99.47% ± 0.97

### 3.12. Robustness

By intentionally enhancing the roughness of the method, small deliberate modifications were made to the chromatographic conditions, such as changing the flow rate (0.1 mL/min), mobile phase composition (2%), and detection wavelength (±2 nm). These variations did not significantly affect the retention time, peak area, or resolution of MTH and DCL. This indicates that the method is robust and capable of withstanding minor operational changes without compromising analytical performance (Table S1).

### 3.13. Solution Stability

During the retention period of 24 and 48 h, standards and samples of MTH and DCL solutions were stored at room temperature and under refrigeration (2°C–8°C). The solutions were analyzed at each time point and compared with freshly prepared samples. No significant changes in the peak area, retention time, or resolution were observed. The %RSD values remained below 2%, indicating that the solutions were stable under the tested conditions (Table S1).

### 3.14. Assay Results of the Dosage Form

Assaying fixed‐dose combination tablets containing MTH and DCL was performed using RP‐HPLC. Multiple batches were analyzed, and the content of each active pharmaceutical ingredient was calculated as a percentage of the labeled claim. Table 3 summarizes the assay results.

**TABLE 3:** Six‐month accelerated testing results of MTH and DCL assays.

Intervals/months	Assay results		Limit
	MTH	DCL	
0	101	100	90%–110%
1	100.2	99.6	
3	99.1	98.3	
6	98.8	97.5	

### 3.15. Dissolution Test Results of the Finished Products

The dissolution tests were conducted using USP Apparatus II (Paddle) at 37.0°C ± 0.5°C and 100 rpm in phosphate buffer (pH 6.8). HPLC analyses were performed at predetermined intervals. MTH and diclofenac sodium showed more than 75% release within 30 min, meeting the USP specifications. Table 4 summarizes the dissolution profiles.

**TABLE 4:** Six‐month accelerated testing results of MTH and DCL dissolution.

Intervals/months	Dissolution results		Limit
	MTH	DCL	
0	97	101	NLT 80% after 30 min
1	95	98	
3	93	97	
6	92	96	

### 3.16. Accelerated Stability Results

The accelerated stability study was performed at 40°C ± 2°C/75% RH ± 5% for 6 months. Samples were analyzed at 0, 1, 3, and 6 months for assay and dissolution. The assay results for DCL K and MTH remained within pharmacopeial limits throughout the study, showing only a slight decrease from initial values (Figure 4). Similarly, dissolution profiles demonstrated minimal variation, with DCL decreasing from 97% to 92% and MTH decreasing from 98% to 93% over the same period. All remain above the acceptance criteria (*Q* ≥ 80%). These findings indicate that the dosage form maintained its potency and release characteristics under accelerated conditions, confirming its stability within the tested timeframe (Tables 3 and 4).

FIGURE 4Assay and dissolution charts for the analyte drugs under accelerated conditions.

**Figure:**
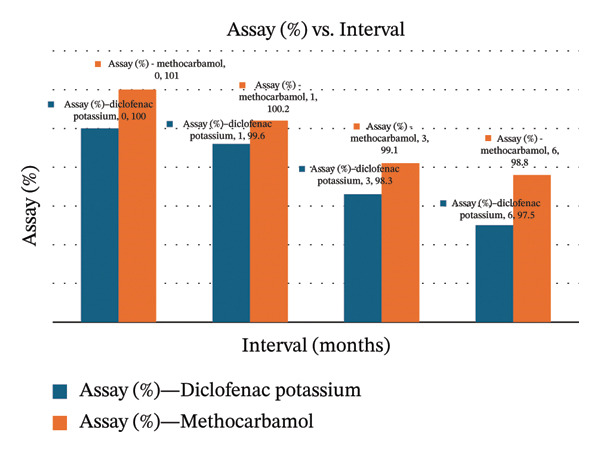
(a)

**Figure:**
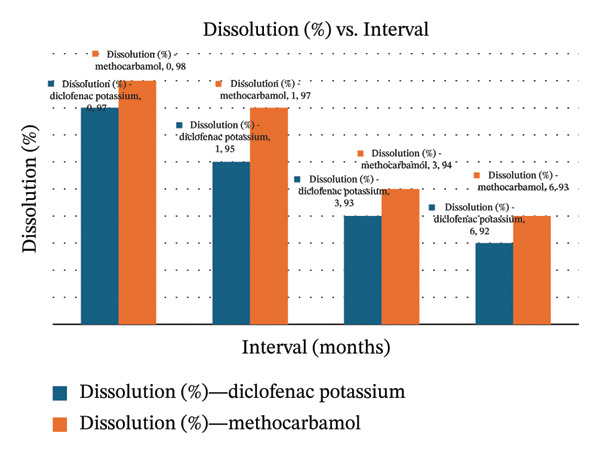
(b)

### 3.17. System Suitability

Chromatographic systems were tested for suitability before sample analysis. In order to evaluate the parameters, we evaluated the theoretical plates, tailing factor, resolution, and %RSD of the peak areas from replicate injections. The results met the predefined acceptance criteria: theoretical plates > 2000, tailing factor < 2, resolution > 2, and %RSD < 2%. According to these findings, the system is capable of reproducible and reliable analysis of MTH and diclofenac sodium (Table 5).

**TABLE 5:** Criteria for selecting an HPLC system for MTH and DCL analysis.

Criteria	MTH	DCL	Limit
Retention time	2.1	6.4	*t*_*R*_ ±10%
Resolution	N/A	19.9	NLT 1.5
Precision	0.1	0.1	NMT 2.0%
Theoretical plates	3408	7390	NLT 2000
Tailing factor	1.0	0.9	NMT 2.0%

### 3.18. Specificity

Specificity was validated by placebo samples, standard solutions, and spiked formulations. This was to guarantee that the method had the ability to correctly quantify MTH and DCL without being affected by excipients or degradation products. The method was specific and suitable for routine analysis; both analytes had well‐resolved peaks with no co‐eluting peaks as observed in chromatograms.

### 3.19. Appraisal of Sustainability Metrics

#### 3.19.1. Eco‐Scale Evaluation

The developed HPLC method was evaluated using the Analytical Eco‐Scale. This is the evaluation of analytical procedures on the basis of the hazards of the reagents used, energy consumption, and waste generation. Ethanol (4 points), phosphate buffer (2 points), energy consumption (1 point), and solvent waste (3 points) were allocated penalty points, making the total 10 penalty points. The score on the Eco‐Scale was 100−9 = 91 (Table 6), which means that the approach is an excellent green analysis (score > 75). This large score shows the approach is environmentally suitable and consistent with GAC. This requires less hazardous solvents and less energy consumption without harming analytical performance (Figure 5).

**TABLE 6:** Points deducted for calculating ESA scores using approved methods.

Parameters	Analytical Eco‐Scale	Penalty points
		HPLC technique
Reagents	Purified water	0
	Potassium dihydrogen phosphate	0
	Ethanol	4

Instruments	Energy for HPLC ≤ 1.0 kWh/sample	1
	Occupational hazard	0
	Ultrasonic	1
	Waste	3
	Total penalty points	9
	Eco‐Scale total score	91

FIGURE 5(a) Bar and (b) pie charts illustrating an Analytical Eco‐Scale evaluation (using illustrative penalty points).

**Figure:**
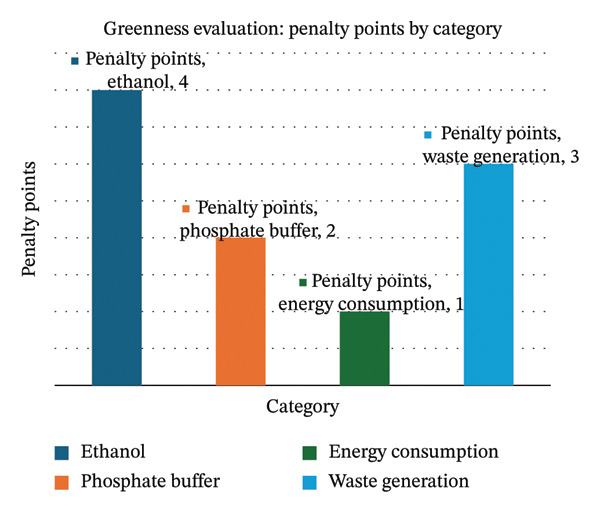
(a)

**Figure:**
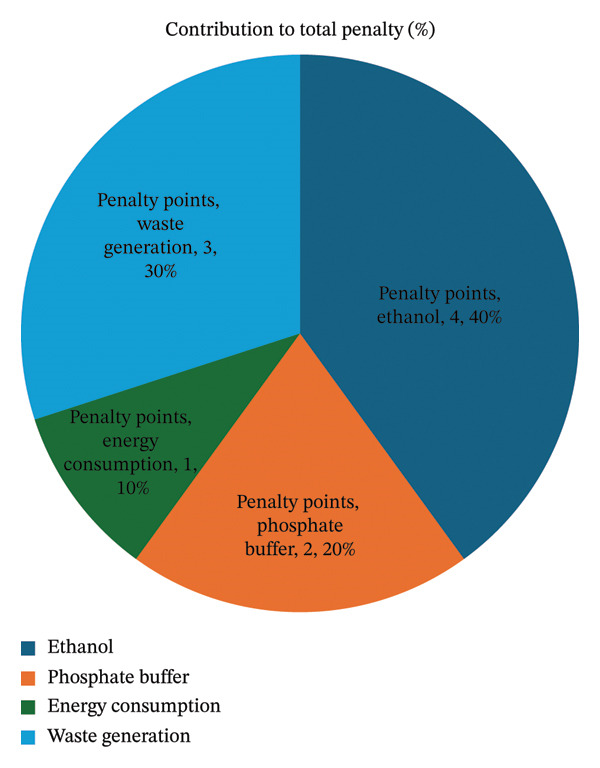
(b)

#### 3.19.2. CACI Evaluation

**FIGURE 6:**
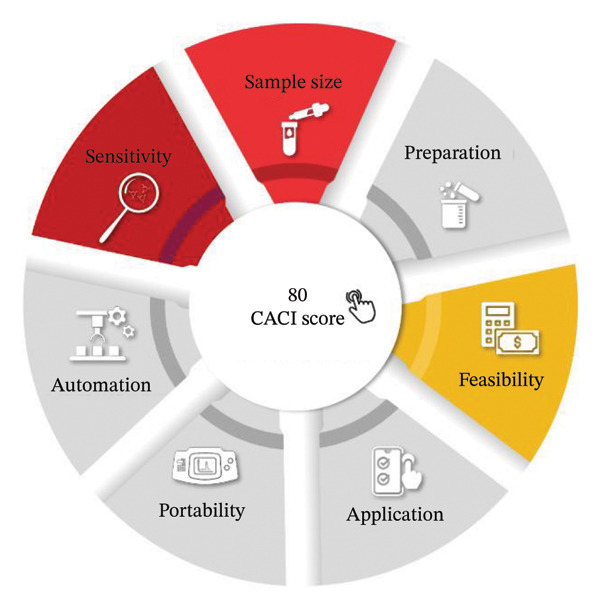
Appraisal of the proposed HPLC method using CACI tool.

The suggested RP‐HPLC technique scored 80 on the CACI (Figure 6), which is high in terms of simplicity, efficiency, and practicality in the CACI system. The high score is a sign of positive characteristics.

Sample size and preparation: The technique only needs under 1 mL of sample and minimal preparation, with preparation taking about 35 min, which is reasonable for routine QC.

Feasibility: The chemicals and reagents used are all commercially available, as are the instruments needed, which are standard in an analytical laboratory. The method is cost‐effective since the cost per sample is less than 10 dollars.

Application: It is a quantitative method that analyzes 2–3 analytes in more than one (> 3) matrix, which makes it more versatile.

Portability and automation: Although the instrumentation is small but not yet portable, the approach provides semi‐automation that enhances throughput efficiently.

Sensitivity and speed: This method has high sensitivity (≤ 1% target concentration) and a short analysis time of 9 min/sample that facilitates quick and high‐quality results (Figure S1).

These features are consistent with the philosophy of click chemistry, namely simplicity and modularity, which makes the method suitable for sustainable and routine pharmaceutical analyses. The inability to be fully portable and fully automated, however, makes it less applicable in field settings. Even with such small constraints, the high CACI score affirms that the technique is creative, environmentally friendly, and workable to complement other conventional validation systems such as ICH Q2(R1).

### 3.20. EPPI Evaluation

**FIGURE 7:**
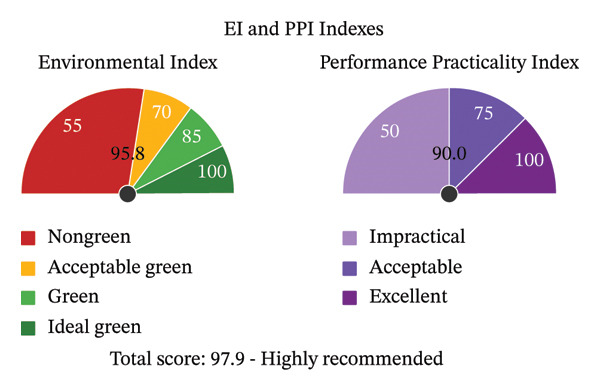
Appraisal of the proposed HPLC method using EPPI tool.

The proposed approach was thoroughly analyzed in terms of the EPPI tool that combines the sustainability of the environment and operational efficiency into a single score. The overall score of the method presented in the developed EPPI report was 97.9 (Figure 7), which puts it in the Highly Recommended category. This high score is an expression of excellent environmental qualities, where the EI is rated at 95.8, which shows a perfect green analysis process. The evaluation of the elements revealed that sample preparation, instrumentation, and waste generation (98.3, 95.0, and 100.0) are incredibly green, and the reagents also achieved high scores (90.0), which also attests to the fact that the environmental burden at each of the workflow levels is minimal. The PPI scored 90.0, which is excellent analysis performance, high applicability of the methods, and high operational feasibility (Figure S2). Together, the EPPI analysis shows that the devised HPLC technique is both capable of meeting and surpassing contemporary demands for a green, efficient, and practical analytical process, aligning with current trends in sustainability within analytical science.

### 3.21. Statistical Comparison With a Reported Method

Using summary statistics (mean ± SD, *n* = 6 per method) and two‐tailed Welch’s *t*‐tests (unequal variances), there were no significant differences in the mean recoveries of the two methods for either analyte (Table S2). The proposed method (99.41 ± 0.80%) and the reported method [15] (99.69 ± 0.96%) for MTH gave *t* = −0.55, df ≈ 9.69, and *p* = 0.60. The F‐test for variances showed that the two methods are equally precise by giving *F* = 1.44 (df = 5,5), *p* = 0.72. For DCL, our method (99.47 ± 0.97%) compared to the reported method [15] (99.13 ± 1.94%) gave *t* = +0.38, df ≈ 7.36, *p* = 0.71; the corresponding F‐test was *F* = 4.00 (df = 5,5), *p* ≈ 0.16, which also showed no statistically significant difference in variances. To sum up, these results indicate that the suggested procedure and the published method are equally accurate and precise for both analytes at *α* = 0.05. Statistical examination using Welch’s *t*‐tests and F‐tests indicated that the mean recoveries for MTH and DCL were similar (*p* > 0.05), as were the variances (*p* > 0.05). This result means that the suggested and reported methods were just as accurate and precise.

### 3.22. A Comparison of Conceptual HPLC With Published Methods

Compared to the methods that have been reported [17–19], the proposed RP‐HPLC has a consistently better sustainability‐performance profile and higher sensitivity (Table S3). The CACI score for the proposed method is 80, which is higher than the scores for the three [17–19] published methods (70, 65, and 67). This means that the proposed method is easier to use and has a more streamlined workflow. The proposed procedure is better for the environment and for operations, with an EI of 95.8% and a PPI of 90.0%. The comparators, on the other hand, have lower PPI values (84.2%, 83.3%, and 83.8%), which means they do not have as good combined performance and practical attributes. The Eco‐Scale scores (proposed 91 vs. 82, 88, and 76) show the same advantage. This is mostly because the mobile phase is a greener phosphate buffer (pH 2.5)–ethanol (30:70, *v*/*v*) instead of systems with sodium dodecyl sulfate/propanol/orthophosphoric acid, acetonitrile, or methanol with triethylamine. The flow rate of 1.0 mL/min (compared to 1.5–2.0 mL/min in some references [18, 19]) and the shorter analysis window help to lower solvent use without lowering resolution. The proposed method has much lower detection and quantification limits (LOD 0.069 μg/mL for MTH and 0.012 μg/mL for DCL; LOQ 0.210 μg/mL for MTH and 0.035 μg/mL for DCL) than other methods that have been reported (1.59/4.83 μg/mL for MTH and 0.44/1.32 μg/mL for DCL [17]; 0.31/0.94 μg/mL for MTH [19]). This shows that it is more sensitive. The conceptual HPLC approach is better than the published methods in terms of EI and analytical performance. This is shown by the greener solvent selection, lower flow/solvent demand, higher overall sustainability scores (CACI, EPPI, Eco‐Scale), and much better LOD/LOQ. This supports its use for routine QC and dissolution applications (all values are shown in Table S3).

## 4. Conclusion

The proposed RP‐HPLC is a straightforward, powerful, and environment‐friendly technique to measure MTH and DCL in fixed‐dose combination tablets. Using assay and dissolution testing in a single validated procedure, it also saves a lot of analysis time, cost, and the use of resources. It is therefore very appropriate for routine quality control. The approach is in accordance with ICH Q2(R1), with high linearity, accuracy, precision, robustness, and specificity. In addition to that, its conformity to the principles of GAC and WAC (with the help of ethanol as a less harmful solvent and its minimal EI) highlights its sustainability. We used the EPPI to test the method. EPPI combines two weighted parts: (i) an EI that measures how green a sample is during preparation and measurement (solvents, waste, hazards, energy/time) and (ii) a PPI that measures how well the method works and how useful it is. EPPI gives a single number score and a pie chart that shows how much of the score comes from environmental (green) and performance/practicality (purple) factors. This gives a WAC view that takes both performance and sustainability into account. On the other hand, the CACI is a tool that focuses on practicality and is based on “click” analytical design. It is usually used to show how easy it is to implement, how modular it is, and how simple it is to use, but it does not fully measure the EI across the workflow. So, EPPI is the best main index for comparing methods, and CACI is also reported to show how useful they are. The high scores of CACI, EPPI, and Eco‐Scale also prove the practicality and receptivity of the method. This makes this approach an effective means of studying pharmaceuticals in the present‐day world. This work helps develop effective, reliable, and environmentally responsible methods of analysis. These methods contribute to the universal transition to sustainability in pharmaceutical quality control.

## Author Contributions

Hedia Ouni and Fahad M. Alminderej: visualization, data validation, and review and editing. Mostafa E. Salem and Sayed M. Saleh: data curation, supervision, and resources. Omkulthom Al kamaly: software, validation, and writing–review and editing. Mahmoud A. Mohamed: conceptualization, method development, data analysis, and manuscript writing. Third‐party involvement: No third‐party individuals or services were involved in the research or manuscript preparation.

## Funding

This research was funded by Princess Nourah bint Abdulrahman University Researchers Supporting Project number (PNURSP2026R917), Princess Nourah bint Abdulrahman University, Riyadh, Saudi Arabia.

## Disclosure

The authors declare that Hikma Pharmaceutical Company had no influence on the design, execution, interpretation, or reporting of the results presented in this study. All authors agreed to be accountable for the content and conclusions of the article.

## Conflicts of Interest

The authors declare no conflicts of interest.

## Supporting Information

Supporting. Figure S1. Evaluation of method sustainability utilizing CACI tool.

Supporting 2. Figure S2. Evaluation of method sustainability utilizing EPPI tool.

Supporting 3. Table S1. Inter‐ and intraday precision, robustness, and stability of solution.

Supporting 4. Table S2. Statistical comparison with a reported method.

Supporting 5. Table S3. A comparison of the conceptual HPLC approach to published methods using ecological assessment metrics and analytical parameters.

## Supporting information


**Supporting Information** Additional supporting information can be found online in the Supporting Information section.

## Data Availability

The data that support the findings of this study are available in this article.
