# Supplementary material for: Eco‐Conscious RP‐HPLC Method for Concurrent Quantification of Assay and Dissolution Testing of Methocarbamol and Diclofenac Potassium in Tablet Formulations
Source: Int J Anal Chem. 2026 Apr 21;2026:8947770. doi: 10.1155/ianc/8947770 (PMC13096726; doi:10.1155/ianc/8947770)
Supplement: Supplementary file 1 — Supporting Information Additional supporting information can be found online in the Supporting Information section. [file IANC-2026-8947770-s001.docx]

**Eco-Conscious RP-HPLC Method for Concurrent Quantification of Assay and Dissolution Testing of Methocarbamol and Diclofenac Potassium in Tablet Formulations**

**Hedia Ouni^1^, Fahad M. Alminderej^2^,** **Mostafa E. Salem^3^, Sayed M. Saleh^2^, Omkulthom Al kamaly ^4^, and Mahmoud A. Mohamed^5^***

^1^ Department of Chemistry, College of Science in Yanbu, Taibah University, Yanbu Governorate, Saudi Arabia; [houniepabdelmlak@taibahu.edu.sa](mailto:houniepabdelmlak@taibahu.edu.sa)

^2^ Department of Chemistry, College of Science, Qassim University, Buraidah 51452, Saudi Arabia; [f.alminderej@qu.edu.sa](mailto:f.alminderej@qu.edu.sa); [e.saleh@qu.edu.sa](mailto:e.saleh@qu.edu.sa)

^3^ Department of Chemistry, College of Science, Imam Mohammad, Ibn Saud Islamic University (IMSIU), Riyadh 11623, Saudi Arabia; [meaSalem@imamu.edu.sa](mailto:meaSalem@imamu.edu.sa)

^4^Department of Pharmaceutical Sciences, College of Pharmacy, Princess Nourah bint Abdulrahman University, P.O. Box 84428, Riyadh 11671, Saudi Arabia; [omalkmali@pnu.edu.sa](mailto:omalkmali@pnu.edu.sa)

*^5^Hikma Pharmaceutical Company, Beni-Suef 62511, Egypt.

*Corresponding author E-mail: [ch.mahmoud88@gmail.com](mailto:ch.mahmoud88@gmail.com); [mmabdelfatah@hikma.com](mailto:mmabdelfatah@hikma.com)

**Abstract:**

A common combination of methocarbamol (MTH) and diclofenac potassium (DCL K) relieves musculoskeletal pain and inflammation through centrally acting muscle relaxants. A dual-function RP-HPLC method was used to analyze the assay and dissolution testing of MTH and DCL K simultaneously. As part of the suggested method, green and white analytical chemistry standards were incorporated to ensure sustainability and compatibility. We performed a chromatographic separation on a C8 column (15 cm × 4.6 mm, 5 µm) with a mobile phase of phosphate buffer and ethanol (30:70 v/v), adjusted to pH 2.5 ± 0.1, yielding sharp and well‑resolved peaks with retention times of 2.183 min for MTH and 6.652 min for DCL. A 275 nm dual UV detector was used to detect the separation at a flow rate of 1.0 mL/min. Sensitivity parameters demonstrated low detection capabilities, with limits of detection (LOD) of 0.069 μg/mL for MTH and 0.012 μg/mL for DCL, and limits of quantification (LOQ) of 0.210 μg/mL and 0.035 μg/mL. Over an extended sample period of 30 minutes with 100 revolutions per minute (RPM), USP Apparatus II (Paddle) was used to measure dissolution at a temperature of 37.0 ± 0.5°C in phosphate buffer (pH 6.8). With both active pharmaceutical ingredients, 75% of the drug was released within 30 minutes. A combination of suitability (RSD 2%) and precision (raw data 2%) makes the method an excellent choice for routine quality control. In keeping with the changing environment and regulatory requirements, pharmaceutical analysis can become more efficient and sustainable. Accelerated stability studies were conducted on the finished dosage form under ICH-recommended conditions for six months. Sustainability was assessed using the Analytical Eco-Scale, Environmental and Practical Performance Index (EPPI), and Click Analytical Chemistry Index (CACI). The method achieved an Eco-Scale score of 91, classifying it as an excellent green method, an EPPI score of 97.9, and a CACI score of 80, confirming its efficiency and practicality for routine quality control.

**Keywords:** Methocarbamol; Diclofenac K; RP-HPLC; Assay and Dissolution testing; Analytical Eco-Scale ; EPPI; CACI; Anti-inflammatory and analgesic effects.


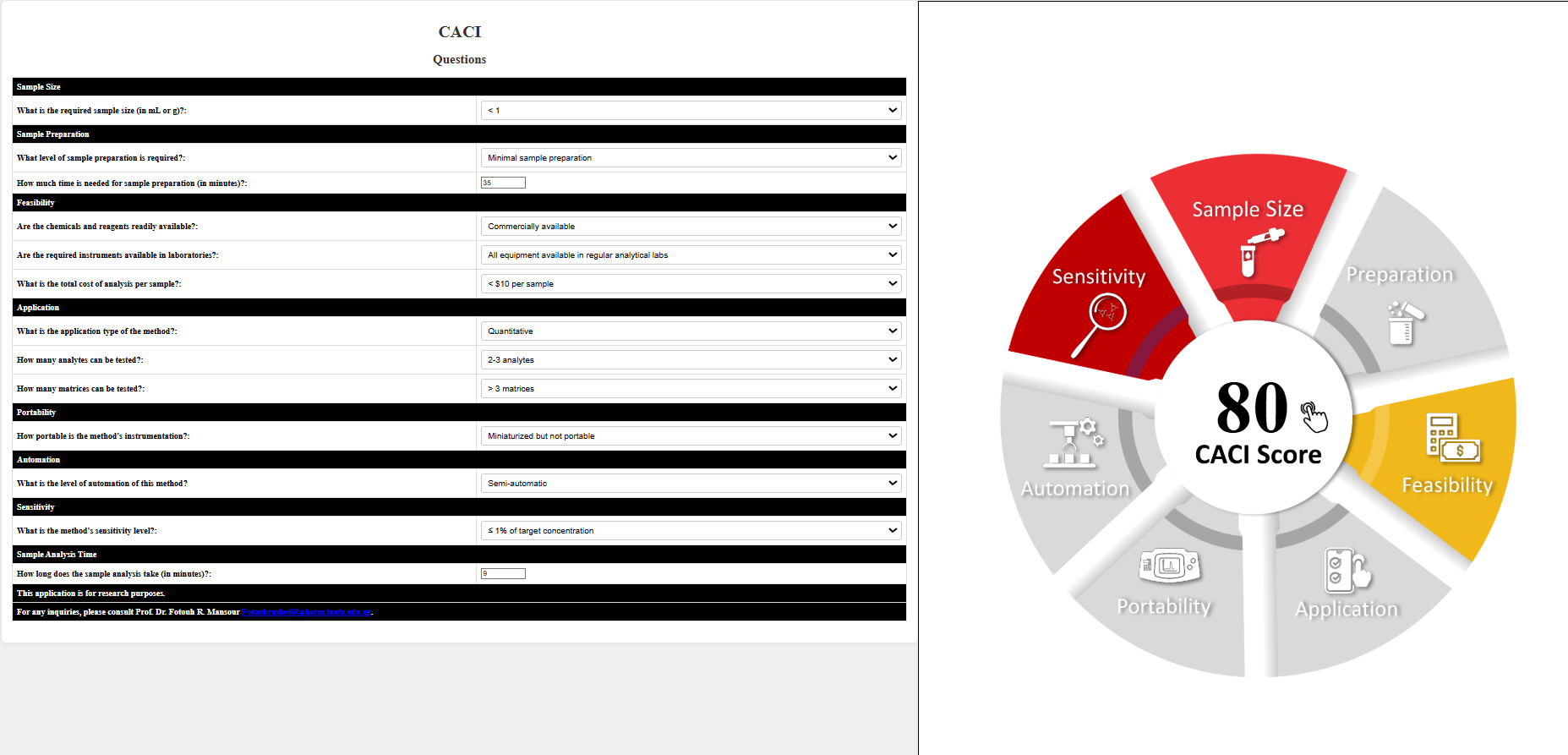


**Figure S1.** Evaluation of method sustainability utilizing CACI tool.


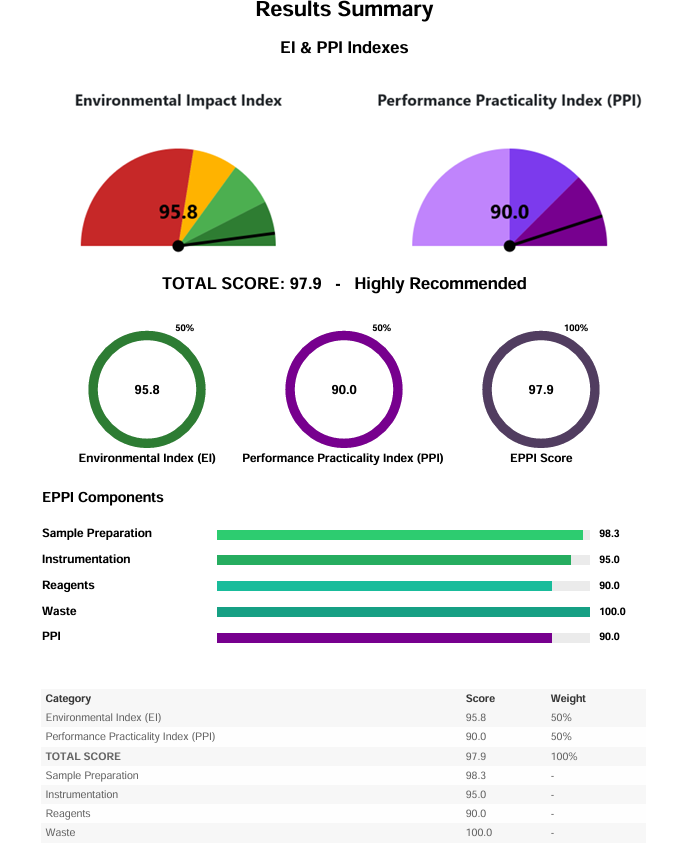


**Figure S2.** Evaluation of method sustainability utilizing EPPI tool.

**Table S1.** Inter and intraday precision, robustness, and stability of solution.

| Parameter | **HPLC** | | **Limit %** |
| --- | --- | --- | --- |
|  | MTH | DCL | **RSD ≤ 2.0%** |
| Day to Day | 0.9% | 07% |  |
| Analyst to Analyst | 0.8% | 0.9% |  |
| Column to Column | 0.7% | 08% |  |
| Flow rate change (±0.1 mL/min) | 0.8% | 0.9% |  |
| pH changes of mobile phase (±0.05) | 0.4% | 0.3% |  |
| Organic Solvent change (±2%) | 0.6% | 0.4% |  |
| Fresh Sample | 0.5% | 0.3% |  |
| Stored Sample in fridge  Stored Sample in autosampler | 0.7%  0.8% | 0.8%  0.9% |  |
| Stored Sample at room temperature | 0.9% | 1.1% |  |

**Table S2.** Statistical Comparison with a Reported Method.

| **Analyte** | **Proposed method (mean ± SD, n)** | **Reported method [15] (mean ± SD, n)** | **Welch t** | **df** | **p (two-tailed)** | **F (var. ratio)** | **F-test p** |
| --- | --- | --- | --- | --- | --- | --- | --- |
| MTH | 99.41 ± 0.80, n=6 | 99.69 ± 0.96, n=6 | -0.55 | 9.69 | 0.5955 | 1.44 | 0.6988 |
| DCL | 99.47 ± 0.97, n=6 | 99.13 ± 1.94, n=6 | 0.38 | 7.35 | 0.7119 | 4.00 | 0.1544 |

**Notes:** Welch t-test compares means without assuming equal variances; F-test compares variances. Alpha = 0.05, two-tailed. Variance ratio F = larger(s²)/smaller(s²) with df1, df2.

**Table S3.** A comparison of the Conceptual HPLC Approach to Published Methods using Ecological Assessment metrics and Analytical Parameters.

| **Items/tools** | **Proposed method** | **Reported method [17]** | **Reported method [18]** | **Reported method [19]** |
| --- | --- | --- | --- | --- |
| **CACI** | 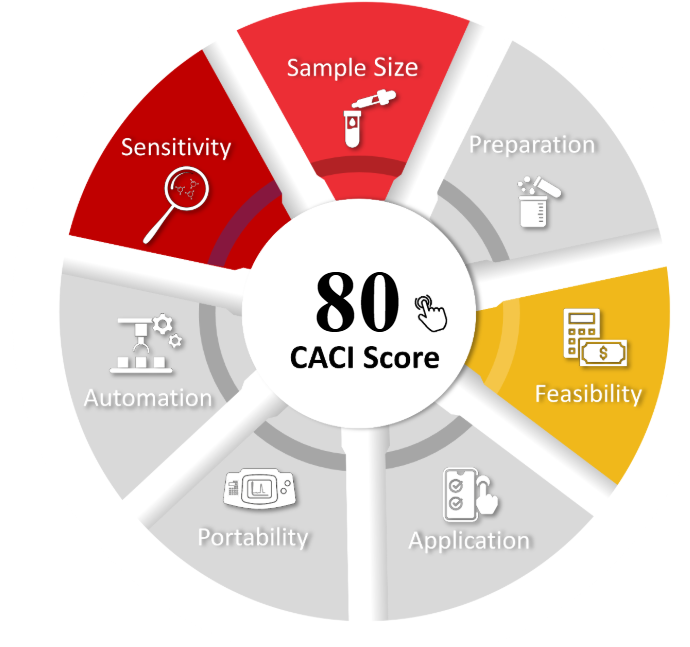 | **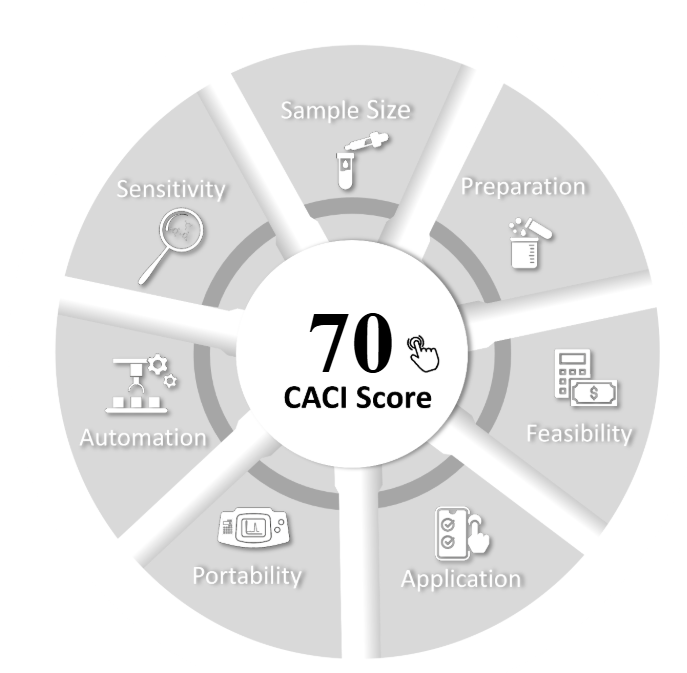** | **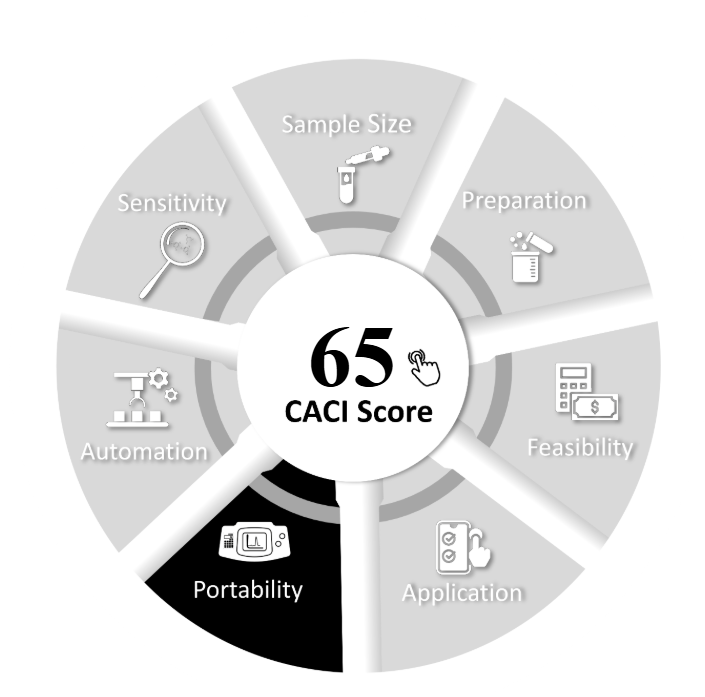** | **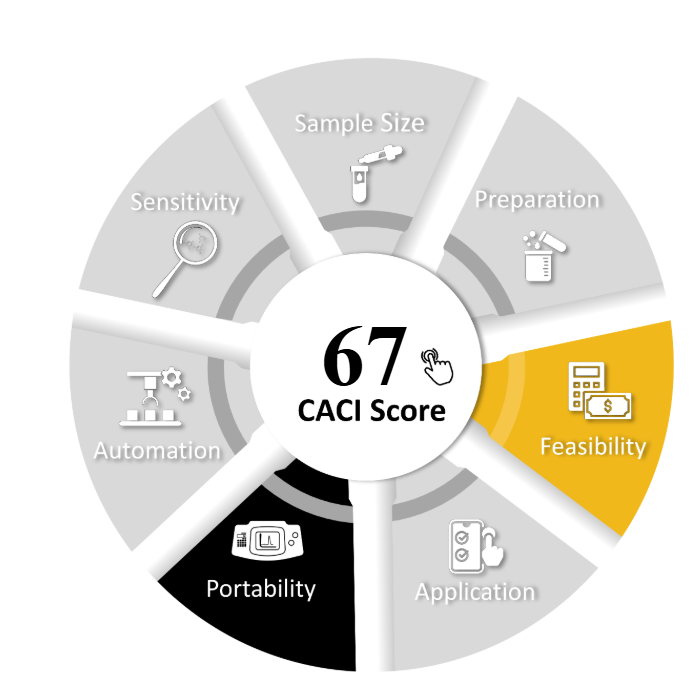** |
| **EPPI** | **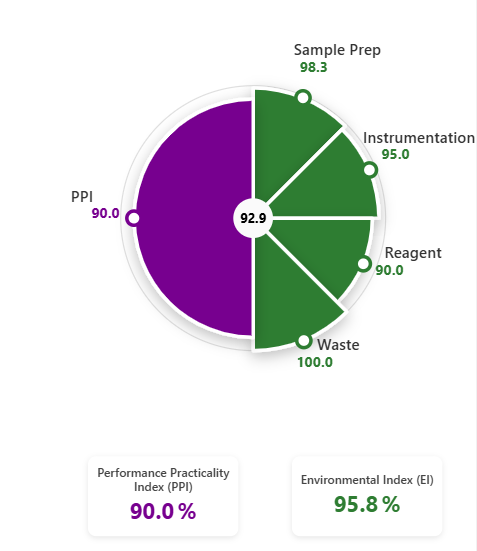** | **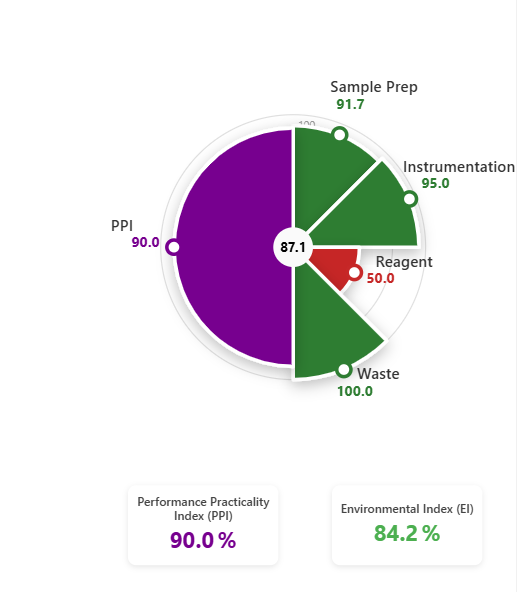** | **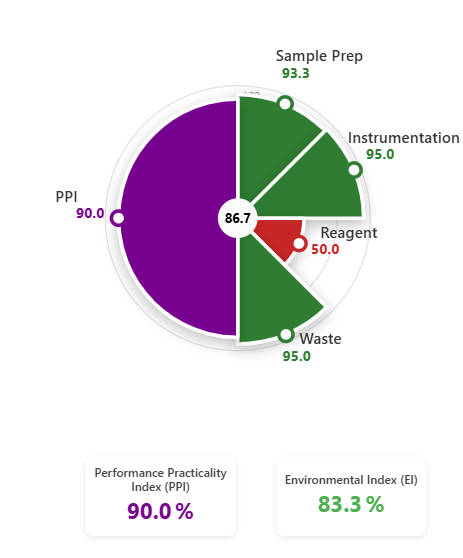** | **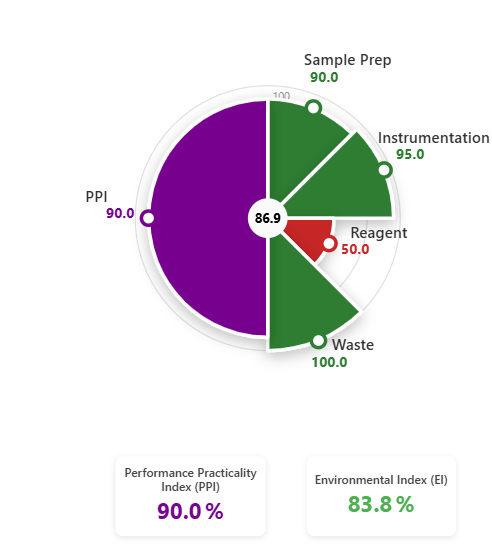** |
| **Eco-Scale** | 91 | 82 | 88 | 76 |
| **Mobile phase** | Phosphate buffer and ethanol (30:70 v/v), adjusted to pH 2.5 ± 0.1 | (0.12 M) [sodium dodecyl sulfate](https://www.sciencedirect.com/topics/pharmacology-toxicology-and-pharmaceutical-science/sodium-dodecyl-sulfate), (15%) n-propanol, 0.02 M [orthophosphoric acid](https://www.sciencedirect.com/topics/chemical-engineering/orthophosphoric-acid), 0.3% [triethylamine](https://www.sciencedirect.com/topics/pharmacology-toxicology-and-pharmaceutical-science/triethylamine), adjusted to pH 6 | 0.05 M KH_2_PO_4_ (pH = 7): acetonitrile (80:30, v/v) | methanol: water: triethylamine (70:30, 0.1% by volume) at pH 3.00 |
| **Flow rate** | 1.0 mL/min | 1.0 mL/min | 1.5 mL/min | 2.0 mL/min |
| **Wavelength** | 275 nm | 220 nm | 220 nm | 254 nm |
| **LOD** | 0.069 μg/mL for MTH 0.012 μg/mL for DCL | 1.59 μg/mL for MTH  0.94 μg/mL for DCL | Not applicable | 0.31 μg/mL for MTH |
| **LOQ** | 0.210 μg/mL for MTH 0.035 μg/mL for DCL | 4.83 μg/mL for MTH  2.86 μg/mL for DCL | Not applicable | 0.94 μg/mL for MTH |
